# Graft-versus-MDS effect after unrelated cord blood transplantation: a retrospective analysis of 752 patients registered at the Japanese Data Center for Hematopoietic Cell Transplantation

**DOI:** 10.1038/s41408-019-0192-x

**Published:** 2019-03-06

**Authors:** Ken Ishiyama, Jun Aoki, Hidehiro Itonaga, Naoyuki Uchida, Satoshi Takahashi, Yuju Ohno, Yoshiko Matsuhashi, Toru Sakura, Makoto Onizuka, Shigesaburo Miyakoshi, Minoko Takanashi, Takahiro Fukuda, Yoshiko Atsuta, Shinji Nakao, Yasushi Miyazaki

**Affiliations:** 1 Department of Hematology, Kanazawa University Hospital, Kanazawa, Japan; 2 Department of Hematology, Yokohama City University Medical Center, Yokohama, Japan; 3 Department of Hematology, Nagasaki University Hospital, Nagasaki, Japan; 4 Department of Hematology, Toranomon Hospital, Tokyo, Japan; 5 Division of Molecular Therapy, The Advanced Clinical Research Centre, The Institute of Medical Science, The University of Tokyo, Tokyo, Japan; 6 Department of Internal Medicine, Kitakyushu Municipal Medical Center, Kitakyushu, Japan; 7 Department of Hematology, Kawasaki Medical School Hospital, Kurashiki, Japan; 8 Leukemia Research Center, Saiseikai Maebashi Hospital, Maebashi, Japan; 9 Department of Hematology and Oncology, Tokai University School of Medicine, Isehara, Japan; 10 Department of Hematology, Tokyo Metropolitan Geriatric Hospital, Tokyo, Japan; 11 Blood Service Headquarters, Japanese Red Cross Society, Tokyo, Japan; 12 Stem Cell Transplantation Division, National Cancer Center Hospital, Tokyo, Japan; 13 Japanese Data Centre for Hematopoietic Cell Transplantation, Nagoya, Japan; 14 Department of Healthcare Administration, Nagoya University Graduate School of Medicine, Nagoya, Japan

## Abstract

Allogeneic hematopoietic stem cell transplantation is the sole curative therapy for myelodysplastic syndrome (MDS). However, there is concern regarding graft failure and relapse in patients who undergo cord blood transplantation (CBT). We conducted a retrospective study of the CBT outcomes in MDS patients using the Japanese Data Center for Hematopoietic Cell Transplantation database. Seven hundred fifty-two de novo MDS patients of ≥18 years of age (median, 58 years) undergoing their first CBT between 2001 and 2015 were examined. Two-thirds of the patients were male, and were RAEB. The cumulative incidences of neutrophil and platelet engraftment at day 100 were 77 and 59%, respectively. The 3-year overall survival (OS) was 41% and the median survival of the patients was 1.25 years. A multivariate analysis of pre-transplant variables showed that the age, gender, cytogenetic subgroups, number of RBC transfusions, HCT-CI and year of CBT significantly influenced the outcome. The cumulative incidence of acute graft-versus-host disease (aGVHD) and chronic GVHD (cGVHD) was 32 and 21%, respectively. A survival benefit was observed in patients who developed cGVHD, but not aGVHD. Our results suggest that CBT is an acceptable alternative graft and that a graft-versus-MDS effect can be expected, especially in patients who develop cGVHD.

## Introduction

Over the long term, there are no effective treatment for the patients with myelodysplastic syndrome (MDS). The outcome of supportive care for higher-risk MDS cases is poor; the prognosis of patients with intermediate-2 and high classifications according to the International Prognostic Scoring System (IPSS) is 1.2 years and 0.4 years, respectively^1^. The use of cytotoxic agents can be considered for MDS subtypes with increased blasts; however, even if complete remission is obtained by combination chemotherapy which is used for the treatment of acute leukemia, the status does not last long, and subsequent event-free survival was not good^2,3^. Even though the launch of new drugs such as hypomethylating agents and multikinase inhibitors has improved the overall survival of MDS patients in recent years, it would be difficult to obtain a cure with these agents^4,5^. Thus, most hematologists recognise that allogeneic hematopoietic stem cell transplantation (allo-SCT) is the sole curative therapy. However, MDS is a disease that most often develops in older people; the median age of onset is 70 years^6^. This means that potential matched-sibling donors are also elderly. Thus, the need for alternative donors for MDS patients is greater in comparison to other hematological diseases. However, Japan has the highest aging rate in the world^7^, which could lead to a shrinking of unrelated volunteer donor pool for allo-SCT, who are currently to be the first choice as an alternative graft source.

Umbilical cord blood transplantation (CBT) represents an alternative graft for patients with no HLA-matched siblings or appropriate unrelated donors. Although the number of CBT procedures is increasing year-by-year^8^, the rates of graft failure and relapse of underlying disease in patients who receive CBT are considered to be higher than those of patients who undergo bone marrow transplantation or peripheral blood stem cell transplantation from unrelated donors, and there have been few large-scale studies on CBT for MDS^9,10^. We therefore conducted a retrospective study to examine the outcomes of MDS patients who received CBT using data obtained from the Japanese Data Center for Hematopoietic Cell Transplantation (JDCHCT) database.

## Methods

### Data collection from the TRUMP

The clinical data on MDS patients of ≥18 years of age who underwent their initial CBT using single CB unit between January 2001 and December 2015 were obtained from the Transplant Registry Unified Management Program (TRUMP) of the JDCHCT^11,12^. Follow-up reports were collected at 100 days, 1 year and annually after CBT using a standardised report form. The following factors were included in the analysis: age at CBT, gender, MDS subtype, cytogenetic subgroup, IPSS classification, performance status (PS), blood type, serological results for HLA-A/B/DRB1, number of RBC and platelet transfusions prior to CBT, type of bridging therapy between the diagnosis and the CBT, effect of bridging therapy, positivity for anti-HLA antibody, hematopoietic cell transplantation-specific comorbidity index (HCT-CI), conditioning regimen, date of CBT, prophylactic agent for graft-versus-host disease (GVHD), date and severity of the development of acute and chronic GVHD, date of relapse, date of last follow-up and survival.

This study was approved as an adult MDS working group study of the Japan Society of Hematopoietic Cell Transplantation (JSHCT) by the committee for Nationwide Survey Data Management of the JDCHCT (study #8-3) and by the ethics committee of Kanazawa University (study #2841).

### Definitions for the analyses

The disease risk was classified into higher-risk MDS, including refractory anemia with excess blasts [RAEB]-1, 2, and lower-risk MDS consisting of the other subtypes of MDS according to the WHO classification^13^. The cytogenetic subgroups were categorized into three risk groups (good, intermediate and poor), which were codified by the International MDS Risk Analysis Workshop^1^ in a central review performed by the adult MDS working group of the JSHCT. The IPSS was classified into higher IPSS risk, consisting of IPSS-high and intermediate-2, and lower IPSS risk, consisting of IPSS-intermediate-1 and low. Bridging therapy was categorized as follows and the number of patients who received each therapy was counted when multiple treatments were performed: combination chemotherapy similar to acute leukemia; low-dose chemotherapy, such as low-dose cytarabine or hydroxyurea; azacitidine; immunosuppressive therapy; and other therapies containing prednisolone, lenalidomide, erythropoiesis-stimulating agents, granulocyte colony-stimulating factor, vitamin D and vitamin K. The effect of the treatment was assessed by the modified IWG criteria^14^. HLA disparity was classified into 3 groups (match, one locus mismatch and ≥2 loci mismatches). The HCT-CI was calculated according to the methods of previous reports^15^. The intensity of the conditioning regimen was classified into 2 groups: myeloablative conditioning (total body irradiation ≥ 8 Gy, busulfan ≥ 9 mg/body, and melphalan ≥ 140 mg) and reduced intensity conditioning, which included other regimens^16^. In vivo T-cell depletion was defined as the use of antithymocyte globulin or alemtuzumab during conditioning. Neutrophil and platelet engraftment were defined by an absolute neutrophil count of >0.5 × 10^9^/L and an absolute platelet count of >20 × 10^9^/L without platelet transfusions within 7 days in 3 consecutive measurements after CBT, respectively. GVHD was diagnosed according to a previous report^15^. Relapse (hematologic, cytogenetic and molecular) was diagnosed in each institution.

### Statistical analyses

The overall survival (OS) was defined as the number of days from CBT until death from any cause. The disease-free survival (DFS) and relapse were evaluated in patients with remission or <5% bone marrow blasts at CBT; the former was defined as the number of days from CBT to relapse. GVHD and relapse-free survival (GRFS) was defined as survival without grade II to IV aGVHD or cGVHD, and without relapse or death from any cause. Non-relapse mortality (NRM) was defined as death without relapse. The cumulative incidence of relapse was examined in patients with remission at CBT and engrafted neutrophils. Any patient who remained alive on the last date of follow-up was censored. The OS rate was calculated using the Kaplan–Meier method and compared using the log-rank test. The cumulative incidence of neutrophil engraftment, NRM and relapse was calculated considering each other type of event as a competing risk and was evaluated using the Fine and Grey test. The following variables were compared in a univariate analysis using Fisher’s exact test: recipient characteristics (age, gender, MDS subtype, cytogenetic subgroup, performance status at the diagnosis, number of RBC and platelet transfusions and type of bridging therapy), donor characteristics (blood type compatibility, gender compatibility, HLA disparity), transplant characteristics (year of CBT, time from the diagnosis to CBT, HCT-CI, intensity of the conditioning regimen, GVHD prophylaxis and in vivo T-cell depletion, nucleated cell count and number of CD34-positive cells of cord blood. To convert from a continuous variable to a binary variable, a median value was used for the threshold. Covariates found to be significant in the univariate analyses (*P* ≤ 0.15) were included in the Cox’s proportional hazards models and Fine and Gray’s proportional hazards models. For both the univariate and multivariate analyses, *P* values were two-sided and *P* values of ≤0.05 were considered to indicate statistical significance. To evaluate the influence of the development of GVHD on the OS, we performed a landmark analysis setting the landmark as day 100 in patients with neutrophil engraftment, and a proportional hazards model treating the development of GVHD as a time-dependent covariate^17,18^. All statistical analyses were performed using EZR (Saitama Medical Center, Jichi Medical University)^19^, which is a graphical user interface for the R software program (The R Foundation for Statistical Computing; http://www.r-project.org, version 3.3.2).

## Results

### Patient characteristics

Seven-hundred and fifty-two patients met the eligibility criteria (Table 1). The age distribution of the patients (grouped by 10 years of age) is shown in Supplementary Fig. 1a. An increase was observed until 70 years of age. The median age of the patients at CBT was 58 years. Two-thirds of the patients were male, with a classification of RAEB. The data of IPSS classification and anti-HLA antibody were missing in 20% of cases. Approximately 95% of the patients received HLA-mismatched CB and >70% received HLA-mismatched CB with the mismatch of ≥2 of 6 HLA loci. The median number of the nuclear cell count was 2.97 × 10^7^/kg. Supplementary Fig. 1b shows the annual number of CBT procedures; the number of CBT procedures rose steadily until 2011. Since then it has been nearly constant. The stratification of the patients by year of transplantation showed that more than half of the procedures were performed in the most recent five-year period (2011–2015). The median period from the diagnosis to CBT was 205 days. The number of patients conditioned with the myeloablative regimen was almost the same as that of patients with non-myeloablative conditioning regimen. Approximately two-thirds of the patients received tacrolimus-based GVHD prophylaxis as a calcineurin inhibitor. In vivo T-cell depletion performed in <5% of the cases.

**Table 1:** Patient characteristics

Factor	Group	*n* = 752 (%)
Age, median (range)		58.0 (18–74)
	<59/≥59	382 (50.8)/370 (49.2)
Gender		
	female / male	262 (34.8)/490 (65.2)
MDS subtypes		
	RCUD	106 (14.1)
	RARS	7 (0.9)
	RCMD	150 (19.9)
	RAEB-1	188 (25.0)
	RAEB-2	272 (36.2)
	RAEB^a^	17 (2.3)
	MDS-U	8 (1.1)
	MDS with isolated del (5q)	4 (0.5)
Cytogenetic subgroups		
	good	302 (40.2)
	intermediate	150 (19.9)
	poor	273 (36.3)
	missing data	27 (3.6)
IPSS		
	Low	31 (4.1)
	Int-1	249 (33.1)
	Int-2	191 (31.4)
	High	138 (25.4)
	missing data	143 (18.4)
Performance status		
	0, 1	654 (87.0)
	2-4	80 (10.6)
	missing data	18 (2.4)
# of RBC transfusion		
	none	71 (9.4)
	1-9	216 (28.7)
	10-19	141 (18.8)
	≥20	214 (28.5)
	missing data	110 (14.6)
# of Platelets transfusion		
	none	149 (19.8)
	1-9	204 (27.1)
	10-19	92 (12.2)
	≥20	197 (26.2)
	missing data	110 (14.6)
Gender compatibility		
	match	309 (41.1)
	mismatch	312 (41.5)
	missing data	131 (17.4)
Blood type compatibility		
	Matched	256 (34.0)
	Minor mismatch	202 (27.0)
	Major mismatch	161 (21.5)
	Major-minor mismatch	129 (17.2)
	missing data	4 (0.5)
HLA disparity		
	matched	45 (6.0)
	1-locus mismatch	169 (22.5)
	≥2-loci mismatch	533 (70.9)
	missing data	5 (0.6)
Anti-HLA antibody		
	positive	144 (19.1)
	negative	408 (54.3)
	missing data	200 (26.6)
HCT-CI		
	0	347 (46.1)
	1	78 (10.4)
	2	68 (9.0)
	≥3	136 (18.1)
	missing data	123 (16.4)
Intensity of conditining Regimen		
	myeloablative	379 (50.4)
	reduced intensity	372 (49.5)
	missing data	1 (0.1)
In vivo T-cell depletion		
	yes / no	36 (4.8)/716 (95.2)
CBT Year		
	2001–2005	85 (11.3)
	2006–2010	242 (32.2)
	2011–2015	425 (56.5)
Time from diagnosis to CBT, median (range)		205 (7–8434)
	≤90 days	123 (16.4)
	91–180 days	209 (27.8)
	>180 days	415 (55.2)
	missing data	5 (0.7)
GVHD prophylaxis		
	Cyclosporine-based	260 (34.6)
	Tacrolimus-based	483 (64.2)
	missing data	9 (1.2)
CB # of NCC x10^7^/kg, median (range)		2.97 (0.80–6.69)
CB # of CD34^+^ cells x10^5^/kg, median (range)		0.81 (0.07–4.76)

*CB* cord blood, *CBT* umbilical cord blood transplantation, *GVHD* graft-versus-host disease, *HCT-CI* hematopoietic cell transplantation-specific comorbidity index, *IPSS* International Prognostic Scoring System, *MDS* myelodysplastic syndromes, *NCC* nuclear cell count

^a^Meet the criteria of RAEB, but cannot distinguish RAEB-1 or RAEB-2 because of insufficient data

### Bridging therapy

Bridging therapy excluding blood transfusions between the diagnosis and CBT was given to 473 of the 752 patients (62.9%). The other 259 patients (63 lower-risk MDS and 196 higher-risk MDS) who did not receive bridging therapy (no bridging cohort) were not evaluated regarding their disease status. The outcomes of the bridging therapy by treatment are shown in Supplementary Table 1. Two-hundred and twenty-nine patients were treated by combination chemotherapy, and 63 (27.5%) obtained CR while 56 (24.4%) responded to the therapy (partial remission [PR]/hematological improvement [HI]). Low-dose chemotherapy and azacitidine, which is the only hypomethylating agent available in Japan, resulted in CR rates of 18.8 and 19.8% and PR/HI rates of 23.8 and 30.6%, respectively. The results of a subgroup analysis classifying patients into lower-risk and higher-risk MDS groups suggested that the overall response rate (ORR), consisting of CR and PR/HI, to combination chemotherapy and low-dose chemotherapy was better in patients with lower-risk MDS than in those with higher-risk MDS (combination chemotherapy, 61.7 vs. 49.4%; low-dose chemotherapy, 62.5 vs. 37.5%), but the ORR to azacitidine was roughly equivalent (48.1 vs. 51.2%).

### Engraftment

**Fig. 1:**
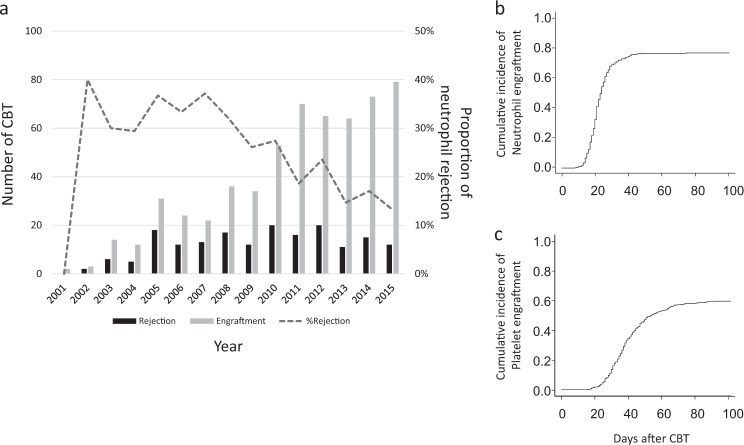
Neutrophil engraftment. **a** Changes in the number and proportion of patients with engraftment. Black bar, annual number of patients with rejection; Gray bar, annual number of patients with engraftment; dashed line, proportion of patients with rejection. **b** Cumulative incidence of neutrophil engraftment. **c** Cumulative incidence of platelet engraftment. *CBT* umbilical cord blood transplantation

Figure 1a shows the number of cases that achieved neutrophil engraftment and developed rejection each year; the rate of rejection is decreasing year by year. The median days of neutrophil and platelet engraftment was 24 and 60, respectively. The cumulative incidences of neutrophil and platelet engraftment on days 30 and 100 were 70.3, 76.7 and 8.4, 59.3%, respectively (Fig. 1b, c). Conversely, neutrophil engraftment was not observed in 58.4% (125/214) of the cases involving patients who died before day 100. The univariate analysis showed that gender, PS, number of RBC transfusions, use of azacitidine and year of CBT were significantly associated with neutrophil engraftment (Table 2a). A multivariate analysis of factors that included these 5 factors revealed the gender (hazard ratio [HR]: 0.78, 95% CI: 0.66–0.92, *P* < 0.003) and year of CBT (HR: 1.12, 95% CI: 0.82–1.54, *P* = 0.48 for the year 2006–2010 and HR: 1.44, 95% CI: 1.07–1.94, *P* < 0.02 for the year 2011–2015, respectively) to be significant factors.

**Table 2:** Clinical factors influencing outcomes

a. Clinical factors influencing neutrophil engraftment.					
Factor	Group	Engraftment (%)	Univariate analysis	Multivariate analysis	
			*P* value	HR (95%CI)	*P* value
Gender			*P* < 0.02		
	female	214/262 (81.7)		1	
	male	363/490 (74.1)		0.78 (0.66–0.92)	*P* < 0.003
Performance status			*P* < 5 × 10^−3^		
	0, 1	514/654 (78.6)		(−)	
	2-4	48/80 (60.0)			
# of RBC transfusion			*P* = 0.09		
	0-19	230/287 (80.1)		(−)	
	≥20	347/465 (74.6)			
Bridging therapy, Azacitidine			*P* < 0.003		
	yes	98/112 (87.5)		(−)	
	no	471/630 (74.8)			
CBT Year			*P* < 8 × 10^−4^		*P* < 0.004^a^
	2001–2005	57/85 (67.1)		1	
	2006–2010	169/242 (69.8)		1.12 (0.82–1.54)	*P* = 0.48
	2011–2015	351/425 (82.6)		1.44 (1.07–1.94)	*P* < 0.02

b. Clinical factors influencing overall survival.					
Factor	Group	3-year OS (95%CI)	Univariate analysis	Multivariate analysis	
			*P* value	HR (95%CI)	*P* value
Age			*P* < 2 × 10^−5^		
	<58	47.7% (42.3–52.9%)		1	*P* < 0.003
	≥59	33.2% (27.8–38.7%)		1.42 (1.13–1.77)	
Gender			*P* < 0.002		
	female	47.1% (40.4–53.5%)		1	*P* < 0.04
	male	37.5% (32.9–42.1%)		1.29 (1.02–1.63)	
MDS subtypes			*P* < 7 × 10^-4^		
	Lower risk MDS^†^	50.6% (43.1–57.6%)		(−)	
	Higher risk MDS^b^	37.0% (32.6–42.4%)			
Cytogenetic subgroup			*P* < 6 × 10^−7^		*P* < 0.001^a^
	good	46.4% (40.2–52.4%)		1	
	intermediate	52.3% (43.2–60.5%)		0.92 (0.66–1.26)	*P* = 0.60
	poor	29.2% (23.6–35.2%)		1.47 (1.16–1.87)	*P* < 0.002
IPSS			*P* < 0.002		
	Lower IPSS	50.4% (43.1–57.3%)		(−)	
	Higher IPSS	36.2% (30.9–41.4%)			
Performance status			*P* < 6 × 10^−7^		
	0, 1	43.1% (38.9–47.2%)		(−)	
	2-4	24.6% (15.4–35.0%)			
# of RBC transfusion			*P* < 2 × 10^−4^		*P* < 0.002^a^
	none	63.4% (50.3–73.9%)		1	
	1-9	46.9% (39.5–53.9%)		1.46 (1.05–2.03)	*P* < 0.03
	10-19	27.2% (19.1–35.8%)		1.88 (1.33–2.66)	*P* < 4 × 10^−4^
	≥20	34.6% (28.0–41.3%)		1.82 (1.31–2.53)	*P* < 4 × 10^−4^
# of Platelets transfusion			*P* < 9 × 10^−4^		
	none	48.3% (38.9–57.0%)		(−)	
	1-9	44.0% (36.7–51.1%)			
	10-19	41.6% (30.9–52.1%)			
	≥20	28.9% (22.3–35.8%)			
Bridging therapy, Azacytidine			*P* = 0.12		
	yes	50.7% (40.1–60.4%)		(−)	
	no	39.7% (35.6–43.7%)			
Bridging therapy, Low-dose chemotherapy			*P* < 9 × 10^−4^		
	yes	24.0% (15.0–34.3%)		(−)	
	no	43.2% (39.1–47.2%)			
Bridging therapy, Combination chemotherapy			*P* = 0.11		
	yes	35.2% (28.5–42.0%)		(−)	
	no	43.4% (38.7–47.9%)			
CBT Year			*P* < 0.003		*P* < 4 × 10^−4a^
	2001–2005	42.6% (31.9–53.0%)		1	
	2006–2010	34.2% (28.2–40.2%)		1.89 (0.88–4.10)	*P* = 0.10
	2011–2015	44.0% (38.4–49.4%)		1.21 (0.56–2.62)	*P* = 0.63
HCT-CI			*P* < 7 × 10^−6^		*P* < 0.006^a^
	0	49.4% (43.6–54.9%)		1	
	1, 2	38.2% (29.6–46.8%)		1.24 (0.95–1.62)	*P* = 0.12
	≥3	25.4% (17.0–34.7%)		1.58 (1.20–2.09)	*P* < 0.002
In vivo T-cell depletion			*P* = 0.052		
	yes	29.6% (15.6–45.0%)		(−)	
	no	41.5% (37.6–45.4%)			

*CBT* umbilical cord blood transplantation, *CI* confidence interval, *HCT-CI* hematopoietic cell transplantation-specific comorbidity index, *HR* hazard ratio, *IPSS* International Prognostic Scoring System, *MDS* myelodysplastic syndromes, *OS* overall survival

^a^overall *P* value

^b^Higher-risk MDS consists of refractory anemia of excess blast (RAEB); lower-risk MDS consists of other MDS subtypes

### Survivals

**Fig. 2:**
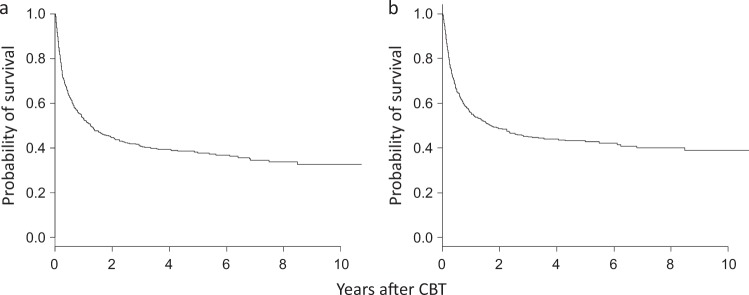
The survival. **a** Probability of the overall survival. **b** Probability of the disease-free survival. *CBT* umbilical cord blood transplantation

The 3-year OS and DFS rates were 40.9 and 45.0%, respectively (95% confidence interval [CI]: 37.0–44.6, 40.7–49.1%, Fig. 2). The median survival of all of the patients and the patients in remission was 1.25 years and 1.71 years, respectively (95% CI: 0.95–1.65 and 1.12–2.61). The univariate analysis of the pre-transplant variables influencing OS showed that age, gender, MDS subtype, cytogenetic subgroup, IPSS, PS, history of RBC and platelet transfusion, low-dose chemotherapy as bridging therapies, year of CBT and HCT-CI were significantly associated with the OS (Table 2b). A subsequent multivariate analysis containing these 11 factors and an additional 3 factors with *P* values of <0.15 (use of azacitidine or combination chemotherapy as bridging therapies and in vivo T-cell depletion) showed that the age (HR: 1.42, 95% CI: 1.13–1.77, *P* < 0.003), gender (HR: 1.29, 95% CI: 1.02–1.63, *P* < 0.04), cytogenetic subgroup (HR: 0.92, 95% CI: 0.66–1.26, *P* = 0.60 for intermediate subgroup and HR: 1.47, 95% CI: 1.16–1.87, *P* < 0.002 for poor subgroup, respectively), number of RBC transfusions (HR: 1.46, 95% CI: 1.05–2.03, *P* < 0.03 for transfusion #1-9, HR: 1.88, 95% CI: 1.33–2.66, *P* < 4 × 10^−4^ for transfusion #10–19 and HR: 1.82, 95% CI: 1.31–2.53, *P* < 4 × 10^−4^ for transfusion # ≥20, respectively), year of CBT (HR: 1.89, 95% CI: 0.88–4.10, *P* = 0.10 for the year 2006–2010 and HR: 1.21, 95% CI: 1.20–2.09, *P* < 0.002 for the year 2011–2015, respectively), and HCT-CI (HR: 1.24, 95% CI: 0.95–1.62, *P* = 0.12 for HCT-CI 1/2 and HR: 1.58, 95% CI: 1.20–2.09, *P* < 0.002 for HCT-CI ≥ 3, respectively) were independently associated with the survival.

### Outcomes according to bridging therapy

Supplementary Table 2a shows the CBT outcomes by bridging therapy. Overall, the patients who received azacitidine had a better prognosis than the patients who did not receive azacitidine, even though the result was not statistically significant (3-year OS rates: 50.7 vs. 39.7%, *P* = 0.12). In addition, the prognosis of patients treated with low-dose chemotherapy was significantly worse than the patients who did not receive low-dose chemotherapy (3-year OS rates: 24.0 vs. 43.2%, *P* < 9 × 10^−4^). The results of a subgroup analysis classifying the patients into lower-risk and higher-risk MDS groups showed that no treatment favourably affected the prognosis in the lower-risk MDS group (3-year OS rate: 55.0%), and the prognosis of patients treated with low-dose chemotherapy tended to be poor (3-year OS rate: 37.9 vs. 53.0%, *P* = 0.093). In the higher-risk MDS group, azacitidine resulted in a significantly better prognosis than the patients who did not receive azacitidine (3-year OS rate: 52.4 vs. 33.2%, *P* < 0.01), while low-dose chemotherapy, immunosuppressive therapy, and other treatments resulted in significantly poor prognostic results (3-year OS rates; 22.9, 17.8, and 30.3%, respectively). Combination chemotherapy did not affect the prognosis of each analysis object, overall patients, lower-risk MDS and higher-risk MDS groups.

### Outcomes according to disease status at CBT

**Fig. 3:**
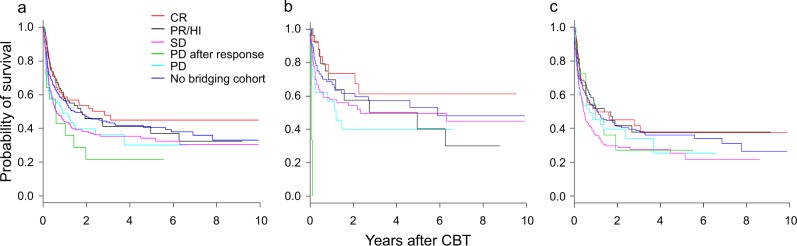
Probability of the OS by status at CBT. **a** All patients. **b** Patients with lower-risk MDS. **c** Patients with higher-risk MDS. *: Higher-risk MDS consists of refractory anemia of excess blast (RAEB; lower-risk MDS consists of other MDS subtypes. Red line, patients in CR; Black line, patients in PR/HI; Magenta line, patients in SD, Green line, patients in PD after response; Cyan line, patients in PD, Dark blue line, patients who did not receive bridging therapy (no bridging cohort). *CBT* umbilical cord blood transplantation, *CR* complete remission, *HI* hematological improvement, *MDS* myelodysplastic syndromes, *OS* overall survival, *PD* progressive disease, *PR* partial remission, *SD* stable disease

Figure 3 shows the correlation between the disease status at CBT and the post-transplantation outcomes. Overall, the patients in CR achieved the best outcomes, with a 3-year OS rate of 49.9% (Supplementary Table 2b), followed by the patients in the ‘no bridging cohort’ and those in PR/HI (3-year OS rates: 44.2 and 40.9%, respectively). Although the CBT outcomes of the patients with SD and PD were almost equivalent (3-year OS rates: 35.3 and 36.1%, respectively), the prognosis of patients with PD after a response was poor (3-year OS rate: 21.4%).

The results of a subgroup analysis classifying patients into lower-risk and higher-risk MDS groups showed that those in CR achieved the best outcomes among lower-risk MDS patients (3-year OS rate: 61.0%), as in the analysis of the entire cohort. However, the outcomes of patients in PR/HI did not appear to be better than those of patients with SD (3-year OS rates: 50.1 and 49.3%, respectively). In addition, the outcomes of patients with PD after remission was extremely poor (3-year OS rate: not reached). In the higher-risk MDS group, the outcomes of patients in CR were relatively good (3-year OS rate: 45.3%), and the patients in PR/HI and with PD showed a similar prognosis (3-year OS rates: 38.1 and 34.2%, respectively). However, the prognoses of the patients with SD and PD after a response were poor (3-year OS rates: 27.8 and 27.3%, respectively). The outcomes of the ‘no bridging cohort’ showed an intermediate outcome between the best prognostic cohort and the worst prognostic cohorts in each analysis object, overall patients, lower-risk MDS and higher-risk MDS groups (3-year OS rates: 44.2, 56.9, and 39.9%, respectively).

### Development of aGVHD

**Fig. 4:**
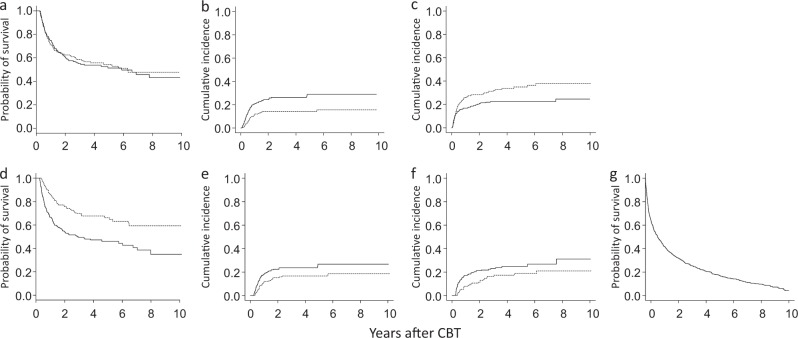
Development of GVHD and the outcome. **a** Development of aGVHD and the survival. Probability of overall survival based on the results of a landmark analysis of patients who were engrafted and who survived for >100 days. Solid line, patients without aGVHD; dotted line, patients with aGVHD. **b** Cumulative incidence of relapse. Solid line, patients without aGVHD; dotted line, patients with aGVHD. **c** Cumulative incidence of non-relapse mortality. Solid line, patients without aGVHD; dotted line, patients with aGVHD. **d** Development of cGVHD and the survival. Probability of overall survival based on the results of a landmark analysis of patients who were engrafted and who survived for >100 days. Solid line, patients without cGVHD; dotted line, patients with cGVHD. **e** Cumulative incidence of relapse. Solid line, patients without cGVHD; dotted line, patients with cGVHD. **f** Cumulative incidence of non-relapse mortality. Solid line, patients without cGVHD; dotted line, patients with cGVHD. **g** Probability of the GVHD and relapse-free survival. *aGVHD* acute graft-versus-host disease, *CBT* umbilical cord blood transplantation, *cGVHD* chronic graft-versus-host disease, *OS* overall survival

The cumulative incidence of aGVHD at day 100 was 32.3% (95% CI: 29.1–35.6%). The landmark analysis to investigate the influence of aGVHD on OS showed that the 3-year OS rates in patients with and without aGVHD were 56.3 and 58.5%, respectively (95% CI: 49.8–62.3% and 50.8–65.4%, *P* = 0.80). The HR for aGVHD, when it was treated as a time-dependent variable, was 1.02 (95% CI 0.80–1.18), *P* = 0.90. The cumulative incidence of relapse in patients who developed aGVHD (14.2%, 95% CI: 9.9–19.3%) was significantly lower than that in those who did not develop aGVHD (26.3%, 95% CI: 21.2–31.7%) (Fig. 4a, *P* < 0.001). In contrast, the cumulative incidence of NRM in patients who developed aGVHD (31.2%, 95% CI: 25.1–37.4%) was significantly higher than that in those who did not develop aGVHD (21.9%, 95% CI: 17.5–26.6%) (Fig. 4b, *P* < 0.01). The univariate analysis showed that PS, HLA disparity and combination chemotherapy as bridging therapy significantly predicted the development of aGVHD, and gender, intensity of conditioning regimen, in vivo T-cell depletion and GVHD prophylaxis had *P* values of <0.15 (Table 3a). In the multivariate analysis of these variables, the PS (HR: 1.78, 95% CI: 1.04–3.06, *P* < 0.04) and GVHD prophylaxis (HR: 0.76, 95% CI: 0.59–0.98, *P* < 0.05) remained significantly associated with the development of aGVHD.

**Table 3:** Clinical factors affecting the development of GVHD

a. Clinical factors affecting the development of aGVHD					
Factor	Group	Development of aGVHD	Univariate analysis	Multivariate analysis	
			*P* value	HR (95%CI)	*P* value
Gender			*P* = 0.12		
	female	36.9%			
	male	31.1%			
Performance status			*P* < 9 × 10^−4^		
	0, 1	34.9%		1	
	2-4	16.5%		1.84 (1.05–3.22)	*P* < 0.04
HLA disparity					
	matched	31.1%	*P* < 0.03		
	1-locus mismatch	24.9%			
	≥2-loci mismatch	36.0%			
Bridging therapy, Combination chemotherapy			*P* < 0.05		
	yes	27.7%		(−)	
	no	35.5%			
Intensity of Conditining Regimen			*P* = 0.10		
	myeloablative	35.9%		(−)	
	reduced intensity	30.3%			
In vivo T-cell depletion			*P* = 0.10		
	yes	19.4%			
	no	33.8%			
GVHD prophylaxis			*P* = 0.072		
	Cyclosporine based	36.9%		1	*P* < 0.05
	Tacrolimus based	30.4%		0.76 (0.59–0.99)	

b. Clinical factors affecting the development of cGVHD					
Factor	Group	Development of cGVHD	Univariate analysis	Multivariate analysis	
			*P* value	HR (95%CI)	*P* value
Age			*P* < 0.002		
	<58	26.8%		(−)	
	≥59	16.8%			
Cytogenetic subgroup			*P* < 0.005		
	good	20.3%		(−)	
	intermediate	32.0%			
	poor	18.3%			
Bridging therapy, Combination chemotherapy			*P* < 0.02		
	yes	16.5%		(−)	
	no	24.4%			
Bridging therapy, Immunosuppressive therapy			*P* = 0.15		
	yes	27.8%		1	
	no	21.0%		1.53 (1.01–2.32)	*P* < 0.05
HCT-CI			*P* < 0.04		
	0	19.9%			
	1, 2	28.3%			
	≥3	16.2%			
In vivo T-cell depletion			*P* = 0.06		
	yes	8.3%		(−)	
	no	22.6%			
Intensity of Conditining Regimen			*P* < 8 × 10^−4^		
	myeloablative	26.9%		1	
	reduced intensity	16.8%		0.59 (0.43–0.81)	*P* < 0.002

*aGVHD* acute graft-versus-host disease, *cGVHD* chronic graft-versus-host disease, *CI* confidence interval, *HCT-CI* hematopoietic cell transplantation-specific comorbidity index, *HR* hazard ratio

### Development of cGVHD and the survival

The cumulative incidence of cGVHD at 1 year was 20.8% (95% CI: 18.0–23.8%). The landmark analysis to investigate the influence of cGVHD on OS showed that the probabilities of 3-year OS in patients with or without cGVHD was 69.7% and 50.5%, respectively (95% CI: 61.5–76.5% and 44.3–56.3%, *P* < 3 × 10^−6^). The 1-year and 3-year GRFS rates were 48.8% and 27.8% respectively (95% CI: 45.1–52.3%, 24.5–31.2%, Fig. 4g). The median GRFS was 0.93 years (95% CI: 0.77–1.16). The HR for the onset of cGVHD, when it was treated as a time-dependent variable, was 0.56 (95% CI: 0.41–0.76, Fig. 4d, *P* < 2.0 × 10^−4^). The cumulative incidence of relapse and NRM in patients who developed cGVHD (16.7 and 16.4%, 95% CI: 11.3–23.1% and 10.8–23.0%, respectively) was significantly lower than in those who did not develop cGVHD (23.5 and 22.4%, 95% CI: 18.5–29.0% and 17.4–27.9%, respectively, Fig. 4e, *P* < 0.03). The univariate analysis showed that age, cytogenetic subgroup, combination chemotherapy as bridging therapy, HCT-CI and intensity of the conditioning regimen significantly predicted the development of cGVHD, and immunosuppressive therapy as bridging therapy and in vivo T-cell depletion had a *P* value of <0.15 (Table 3b). In a multivariate analysis of factors that included these variables, immunosuppressive therapy (HR: 1.47, 95% CI: 1.03–2.09, *P* < 0.04), and the intensity of the conditioning regimen (HR: 0.65, 95% CI: 0.44–0.83, *P* < 0.002) were the variables that remained significantly associated with the development of cGVHD.

## Discussion

CBT have undergone rapid development since it received insurance approval in 1998 in Japan^3,20^. JSHCT guidelines currently revised in 2018 recommend selecting CB or an HLA-1 allele-mismatched unrelated donor or an HLA-1 allele-mismatched related donor in case no matched siblings or unrelated donors are available. SCT from HLA-haploidentical relatives has only been performed as clinical trials, and has not been generalised yet. CBT was chosen by physicians when a CB graft that was 4–6 HLA-serotype compatible with their patients and contained nucleated cells more than 2.0 × 10^7^/kg per recipient’s body weight. The current study revealed that the number of CBT procedures for MDS has been increasing year by year, and that the number of CBT procedures is correlated with an increase in patient age. Graft rejection occurred in approximately 25% of cases, which was higher in comparison to other graft sources^21,22^ and previous reports on CBT for hematological maliganancies^9,23^. However, the rate of graft rejection has tended to decrease year by year, and has declined to <15% in 2015. As anti-HLA antibody-positive recipients are known to have an increased risk of rejection, screening for anti-HLA antibodies has recently been carried out at the time of transplantation^24,25^. Although the anti-HLA antibody data were missing in 20% of cases in this study, avoiding CB grafts that are recognised by recipients’ anti-HLA antibodies may have contributed to the reduction of the rejection rate.

Although the engraftment failure rate in CBT is being improved year by year, the rate in 2015 (13/81, 13.8%) is still high and may make physicians hesitate to choose CBT for patients with MDS. The rate of engraftment failure was similar between the patients who were exposed to chemotherapy for bridging therapy and ‘no bridging cohort’. This study revealed the significantly lower rate of engraftment failure in patients who received CB grafts, of which mismatched HLA with the recipient was less than one, and those containing nucleated cells ≥ 2.0 × 10^7^/kg^26^ with CD34^+^ cells ≥ 0.8 × 10^5^/kg. Choosing these favorable grafts may be useful for ensuring engraftment of CB in patients with MDS.

Among three different regimens as bridging therapy, intensive combination chemotherapy was more effective in inducing CR of MDS before CBT than azacitidine or low-dose chemotherapy. However, the beneficial effect of combination chemotherapy did not lead to a better outcome of CBT probably due to increased organ toxicities associated with intensive chemotherapy. There was no significant difference in the ORR rate after CBT among the three different group of this study cohort. Recent studies showed the lower rate of transplant-related toxicity and a better long-term prognosis after allo-SCT in MDS patients treated with azacitidine^27,28^. The beneficial effect of azacitidine may be offset by its weaker immunosuppressive effect than other chemotherapies, which potentially leads to increased incidence of engraftment failure in CBT recipients.

The factors that contributed to OS, such as the age, MDS subtypes and cytogenetic subgroup were almost the same as those reported in previous studies^9,29–34^. However, it is noteworthy that the number of RBC transfusions was extracted as a factor that adversely affected OS. The negative effect of transfusions is most likely attributed to iron overload though we could not prove its causal relationship with poor prognosis, due to the lack of the data on iron metabolism including serum ferritin levels in TRUMP database. The prognostic impact of iron chelate therapy before transplantation on the outcome of CBT is a subject of future studies.

In the lower-risk MDS group, the 3-year OS rate of patients with SD at CBT was about 50%. In contrast, the prognosis of PD cases that temporarily responded to bridging therapy was extremely poor, although the number of cases was low. In the higher-risk MDS group, the 3-year OS rate of 59 patients in CR at CBT and 77 patients with PR/HI was 45.3 and 38.1%, and about 27% of the 146 patients with SD or PD with temporary responded to the bridging therapy. The efficacy of CBT may thus be limited for patients who were refractory to bridging therapy.

Although the patients who developed aGVHD showed a low rate of recurrence, the benefit was offset by the high rate of TRM. In contrast, the development of cGVHD lowered the rate of recurrence without increasing the incidence of TRM, leading to better OS. Similar beneficial effects of cGVHD after SCT have been reported by Kroger et al.^35^ and Narimatsu et al.^36^ Mild cGVHD after CBT may produce an antitumor effect without causing severe organ damages and thereby improve survival. On the other hand, poor PS at CBT was extracted as a significant risk factor for aGVHD. Factors that lower PS, such as infections and advanced diseases may make patients susceptible to aGVHD.

One limitation of this study is that the TRUMP database did not include blood cell counts at the time of the diagnosis. Thus, we could not incorporate the IPSS or revised IPSS (IPSS-R) into the analysis^1,9,37^, which would likely be important prognostic factors^30,38^. Although the result of IPSS classifications was collected by the TRUMP, approximately 20% of data were missing; therefore, the MDS subtype was used as a factor contributing to the OS instead of IPSS. Since the data items collected by TRUMP are currently being revised by JDCHCT, we plant to incorporate IPSS into our analysis in the next analysis. Another major problem is that the TRUMP database did not include data on the effectiveness of different treatments given to patients. Thus, in patients who underwent multiple treatments, we were unable to determine which treatment was effective. For these reasons, we only evaluated the outcomes of each treatment described in the database. As recent studies revealed prognostic values of somatic mutations including *TP53* in MDS patients undergoing allo-SCT^39,40^, the data of clinical sequencing needs to be collected by JDCHCT.

Our results suggest that CBT is acceptable as an alternative SCT procedure for MDS patients and is associated with the graft-versus-MDS effect. For patients with high-risk MDS, CBT may be a preferable choice of allo-SCT procedure based on the graft-versus-MDS effect associated with cGVHD. To further improve the outcome of CBT for MDS, it is essential to develop more effective bridging therapy with less toxicities than conventional therapies to control high-risk MDS, and perform CBT for transfusion-dependent patients at an early stage before developing iron overload.

## Supplementary information

Supplemental figure 1

Supplemental table 1

Supplemental table 2
