# Supplementary material for: Graft-versus-MDS effect after unrelated cord blood transplantation: a retrospective analysis of 752 patients registered at the Japanese Data Center for Hematopoietic Cell Transplantation
Source: Blood Cancer J. 2019 Mar 6;9(3):31. doi: 10.1038/s41408-019-0192-x (PMC6403210; doi:10.1038/s41408-019-0192-x)
Supplement: Supplementary file 1 — Supplemental figure 1 [file 41408_2019_192_MOESM1_ESM.pptx]

## Slide 1
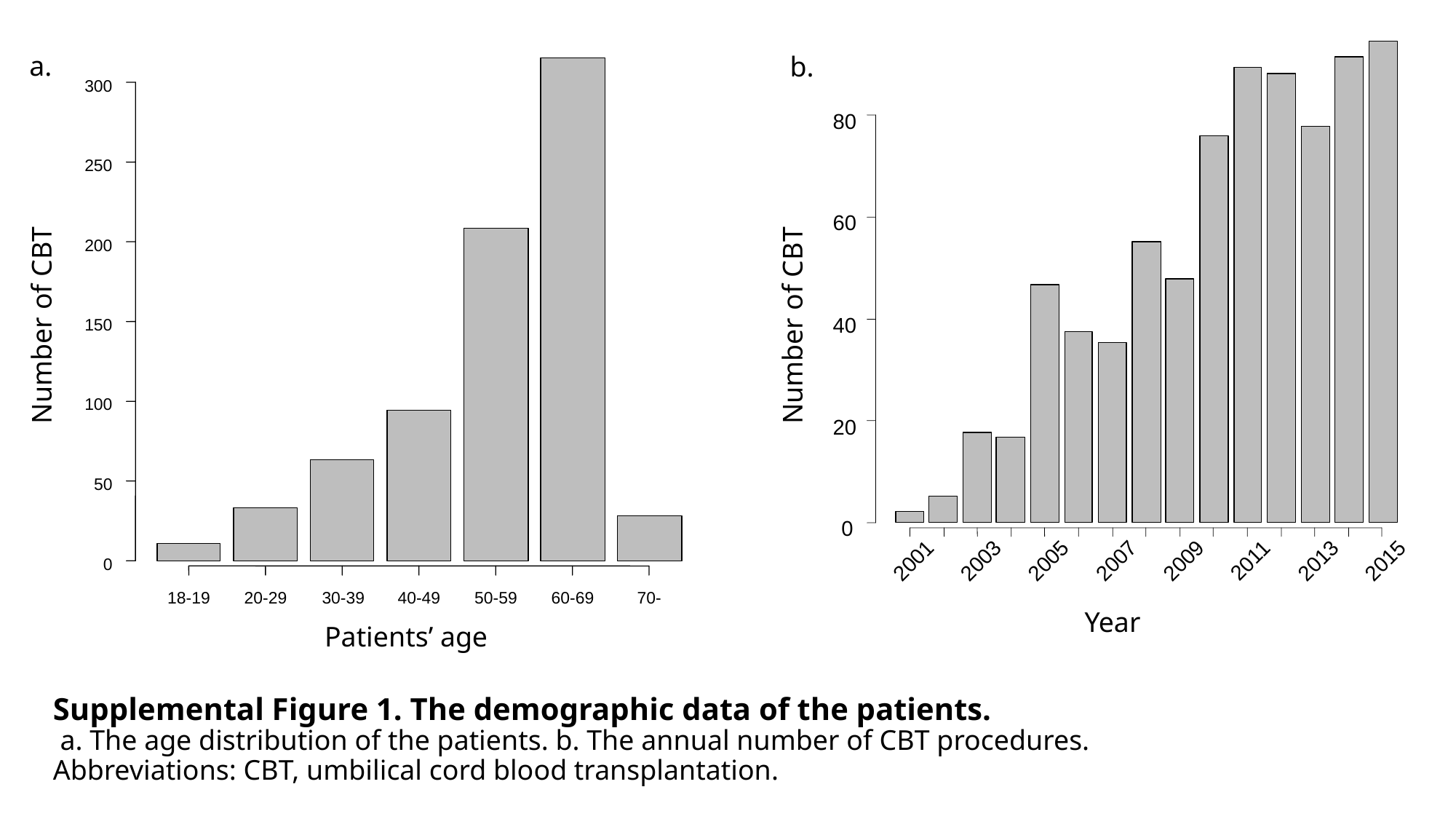

300
250
200
150
100
50
0
18-19
20-29
30-39
40-49
50-59
60-69
70-
a.
b.
80
60
40
20
0
2001
2003
2005
2007
2009
2011
2013
2015
Number of CBT
Number of CBT
Year
Patients’ age
# Supplemental Figure 1. The demographic data of the patients. a. The age distribution of the patients. b. The annual number of CBT procedures. Abbreviations: CBT, umbilical cord blood transplantation.
