# Supplementary material for: Graft-versus-MDS effect after unrelated cord blood transplantation: a retrospective analysis of 752 patients registered at the Japanese Data Center for Hematopoietic Cell Transplantation
Source: Blood Cancer J. 2019 Mar 6;9(3):31. doi: 10.1038/s41408-019-0192-x (PMC6403210; doi:10.1038/s41408-019-0192-x)
Supplement: Supplementary file 3 — Supplemental table 2 [file 41408_2019_192_MOESM3_ESM.pdf]

Supplemental Table 2. CBT outcomes by bridging therapy and status at CBT.

a. Outcomes by bridging therapy.

Factor	Group	All patients 3-year OS (95%CI)	All patients P value	Lower-risk MDS* 3-year OS (95%CI)	Lower-risk MDS* P value	Higher-risk MDS* 3-year OS (95%CI)	Higher-risk MDS* P value
Combination chemotherapy			P=0.11		P=0.34		P=0.33
	yes	35.2% (28.5-42.0%)		43.3% (27.7-57.9%)		33.2% (25.8-40.7%)	
	no	43.4% (38.7-47.9%)		52.1% (43.6-60.0%)		39.3% (33.7-44.7%)	
Low-dose chemotherapy			$P<9x10^{-4}$		P=0.093		P<0.007
	yes	24.0% (15.0-34.3%)		27.9% (9.1-50.7%)		22.9% (13.1-34.4%)	
	no	43.2% (39.1-47.2%)		53.0% (45.0-60.3%)		39.2% (34.4-43.9%)	
Azacitidine			P=0.12		P=0.25		P<0.02
	yes	50.7% (40.1-60.4%)		45.2% (23.6-64.7%)		52.8% (40.7-63.5%)	
	no	39.7% (35.6-43.7%)		51.8% (43.8-59.2%)		34.7% (30.0-39.4%)	
Immunosuppressive therapy			P=0.70		P=0.65		P<0.02
	yes	37.7% (27.7-47.7%)		52.6% (38.1-65.2%)		17.8% (7.5-31.7%)	
	no	41.5% (37.4-45.6%)		49.7% (40.7-57.9%)		39.0% (34.3-43.6%)	
Others			P=0.89		P=0.53		P=0.14
	yes	39.7% (31.2-48.0%)		52.3% (38.5-64.3%)		30.3% (20.0-41.3%)	
	no	41.2% (36.9-45.4%)		50.1% (41.0-58.6%)		38.3% (33.4-43.1%)	
No intensive therapy		45.0% (38.7-51.1%)		55.0% (41.9-66.4%)		41.5% (34.3-48.5%)	

b. Outcomes by status at CBT.

Factor	All patients n	All patients 3-year OS (95%CI)	All patients P value<0.03 †	Lower-risk MDS* n	Lower-risk MDS* 3-year OS (95%CI)	Lower-risk MDS* P value $<2x10^{-5}$ †	Higher-risk MDS* n	Higher-risk MDS* 3-year OS (95%CI)	Higher-risk MDS* P value=0.07 †
CR	85	49.9% (37.8-60.8%)		26	61.0% (36.0-78.8%)	$P<2x10^{-6}$	59	45.3% (31.6-58.1%)	
PR/HI	101	40.9% (29.0-52.5%)		24	50.1% (25.8-70.3%)	$P<2x10^{-8}$	77	38.1% (24.4-51.6%)	
SD	201	35.3% (28.4-42.2%)		66	49.3% (36.1-61.2%)	P<0.001	135	27.8% (20.1-36.0%)	
PD after remission	14	21.4% (5.2-44.8%)		3	NE	P=0.09	11	27.3% (6.5-53.9%)	
PD	74	36.1% (23.7-48.6%)		21	39.7% (18.6-60.2%)	$P<1x10^{-4}$	53	34.2% (19.2-49.8%)	
Not evaluated ‡	259	44.2% (37.8-50.5%)		63	56.9% (43.2-68.5%)		196	39.9% (32.6-47.1%)	

*: Higher-risk MDS consists of refractory anemia of excess blast (RAEB); lower-risk MDS consists of other MDS subtypes.

†: overall P value.

‡: Patients who did not receive intensive bridging therapy (='no bridging cohort') were not evaluated for the effect.

Abbreviations: CBT, umbilical cord blood transplantation; CI, confidence interval; CR, complete remission; HI, hematological improvement; HR, hazard ratio; NE, not evaluable; OS, overall survival; PD, Progressive Disease; PR, partial remission; SD, stable disease.
